# Well-being approaches in public health … what’s new?

**DOI:** 10.17269/s41997-026-01191-0

**Published:** 2026-04-09

**Authors:** Lindsay McLaren

**Affiliations:** University of Calgary, Calgary, AB Canada

The last few years have seen a flurry of activity around “well-being” approaches in public health. Recent examples in Canada include the 2025 Chief Public Health Officer’s report, *Working together to thrive: well-being and public health* (PHAC, 2025), and the Public Health Association of British Columbia’s 2025 Summer Institute and ongoing webinar series focused on *Well-being societies* (PHABC, n.d.).

In the preamble to its 1946 constitution, the World Health Organization (WHO) defined health as “a state of complete physical, mental and social **well-being** and not merely the absence of disease or infirmity” (WHO, n.d., emphasis added). A focus on well-being is therefore nothing new. What are we to make of this new popularity, and what kinds of opportunities and challenges does it bring for our shared vision of supporting the public’s health in this time of polycrisis?

The current enthusiasm around well-being must be situated within growing international momentum around a *well-being economy.* As discussed by the Wellbeing Economy Alliance (est. 2018), a collaboration of individuals and organizations working together to transform the economic system, a well-being economy is:

… an economy designed to serve people and the planet, not the other way around. Rather than treating economic growth as an end in and of itself and pursuing it at all costs, a Wellbeing Economy puts our human and planetary needs at the centre of its activities, ensuring that these needs are all equally met, by default. (WEAll, n.d.)

The “economy” part is important for at least three reasons. First, it draws attention to the globally dominant neoliberal capitalist paradigm, which drives social inequality and environmental devastation (social and ecological determinants of health). Neoliberal political economy is grounded in economic models that assume infinite growth (Ghosh, 2021). On a finite planet, those models are patently unviable. Yet their hegemony results in the ongoing and increasingly aggressive pursuit of a narrow vision of economic growth via policies of deregulation, austerity, privatization, trade liberalization, etc. that have profoundly harmful and unfair social and ecological consequences. Fundamentally premised in competition and exploitation of people and of the earth, this paradigm intersects with racism, sexism, ableism, and other forms of systemic discrimination to create highly unequal, and avoidable, health damage across the lifespan (McGibbon, 2025).

Second, well-being *economy* is important because it invites us to reclaim the word *economy* in very important ways. Neoliberal capitalism has been normalized and its role in causing immense harms is thus obscured. Indeed, “there is no alternative,” to austerity budgets and tax cuts, is the slogan that gave neoliberalism its broad legitimacy. In the Canadian context, there were deliberate efforts to lower public expectations of government and to increase political apathy (Himelfarb, 2024). “The economy” is, moreover, made out in mainstream discourse to be fixed, external, and restricted to technical experts (Stanford, 2015). The well-being economy frame and movement invites us to consider, instead, that “the economy” is simply *how we produce and provide for one another* or, even more simply, *how we care for one another* (WEAll, n.d.). We are the economy; and recognizing this can be empowering.

Third, and significantly, the well-being economy frame creates space to envision equity-centered alternatives. The contours of alternatives start to become visible when we realize that they are the *opposite* of those under neoliberal capitalism. For example, whereas the current political economic paradigm is premised on competition, scarcity, and exploitation as ways to organize society, alternative premises, drawn from Indigenous wisdom and leadership, could (or should) include equity, collectivity, reciprocity, and accountability to each other and to all living things (PHABC, 2026).

If we are concerned about the public’s health including health equity, we must engage with the profoundly harmful political economic paradigm, including who benefits from such arrangements (McLaren & McGibbon, 2025). How are “well-being approaches” in public health faring in this regard?

So far, it would be fair to say, not very well, or not at all. One illustration is the *WHO Geneva Charter for Well-being* (WHO, 2021), which aspires to “well-being societies.” Building on the 1986 Ottawa Charter for Health Promotion and subsequent global conferences, the Geneva Charter describes “well-being societies” as those that are “committed to achieving equitable health now and for future generations without breaching ecological limits.” Indeed, it is difficult to argue with such a vision. However, the Charter is silent on the economic growth paradigm, even though that paradigm is incompatible with its own vision of equity within ecological limits. Replacing “economy” with the more nebulous “society” furthermore risks diluting or diverting our attention away from the ways in which entrenched and far-reaching tentacles of capitalism shape nearly every aspect of our lives.

A second illustration is the recent WHO European Region Primer on *Unlocking funding for well-being, equity and healthy societies* (WHO, 2026), which not only doesn’t question the economic growth paradigm but actively supports it. It does so by, for example, aspiring to “inclusive economic growth” and aiming to “balance” various “well-being capitals,” including human, social, economic, and environmental. While it is true that governments can do a better or worse job of redistributing economic growth (Osberg, 2021), it is abundantly clear that “economic growth at any cost fails us all” (De Schutter, 2026). Phrases like “inclusive growth” and “sustainable growth” are thus oxymorons and should be called out as such. Moreover, using the word “capital,” which according to most definitions has to do with private monetary wealth, to describe people and the earth, should prompt serious pause.

What are some ways that we could embrace, or at least meaningfully engage with, the radical intent of the *well-being economy* frame? A first step is to engage *critically* with capitalism and the economic growth paradigm. This includes resisting the trope that there can be “win-win” arrangements where both health and the capitalist economy can benefit (McLaren & Mykhalovskiy, 2024). Engaging in this manner is challenging because it requires the humility to recognize that many of us in public health benefit from these harmful structures as well-paid professionals and/or because our area of work—such as clinical medicine or epidemiology—epistemically aligns with economic liberalism and that is what gives us power and status.

A second way to engage constructively is to ground ourselves in “the economy” understood as *how we produce and provide for one another*, and to work together to articulate the contours of an equity-centered alternative to the current paradigm. The Wellbeing Economy Alliance offers four principles to guide us in doing so (WEAll, n.d.):

***Pre-distribution***: Rather than leaving it to people to fend for themselves or relying on limited redistributive mechanisms, we come up with ways to *pre-distribute* power, wealth, time, and income so that the heavy lifting is done by the economy itself.

- This invites us to appreciate Canada’s formerly robust tax/transfer redistribution mechanisms developed as part of the welfare state (largely eroded under neoliberalism), while recognizing the need to exercise different—and better—ways to share power that foreground all people and planet. Examples include cooperative and worker-owned organizations and businesses, community wealth building initiatives, normalization of living wages, and building and strengthening bioregional economies which embody reciprocity and kinship with the earth.

***Purpose***: The purpose of the economy becomes exclusively to deliver human and ecological well-being.

- This includes important ongoing work to “measure what matters,” such as the federal government’s Quality of Life framework (Government of Canada, 2025), but it also goes far beyond that to empower the critical narrative shift required to ensure that “what matters” *replaces* GDP and meaningfully guides public policy at different levels.

***Prevention***: Rather than fixing the harm we do to nature and people, we adopt preventive measures that stop harm from happening in the first place.

- Examples include continuing to speak up for strong regulatory approaches to reduce the health damaging effects of harmful corporate sectors like fossil fuels, food, and finance, while recognizing that limits to corporate power will always be constrained in a growth-dependent economy (Puerto, 2026). It also includes advocating for essential services and supports to be in the *public* rather than private sector, to ensure that they serve the public good rather than the interests of a privileged minority.

***People-powered***: Economic decisions are powered by the people, who become directly involved in decision making and agenda setting.

- This involves public education, forums, and spaces to support people in replacing the toxic hegemony of “the economy” as fixed, external, and exclusionary, with a meaningful understanding that *we are the economy* and we can therefore shape it so that it serves all people and the planet rather than the other way around. Examples include citizen assemblies and participatory budgeting (e.g., CCPA, n.d.).

With respect to replacing the organizing principles of capitalism—e.g., competition, scarcity, suffering—with alternatives—such as collectivity, reciprocity, and accountability, one source of inspiration is Thurston’s (brown & Thurston, 2024) reimagining of the word “citizen” as a verb, which supports collective power. “To citizen,” according to Thurston, means:

- To show up and assume that you have a role to play
- To understand power and to be literate in it
- To commit to the collective self and not just the individual self
- To invest in relationships, with self, with others, and with the planet.

The *well-being economy* frame and movement thus advance “well-being” approaches in very important ways and present an exciting opportunity for public health actors in different roles and spaces to come together around our shared vision of supporting the public’s health. We warmly welcome ongoing, respectful dialogue in this space, including through (hint, hint) the journal’s standing call for submissions on *Ecological dimensions of public health and a well-being society* (CJPH, n.d.).

Il y a depuis quelques années un tourbillon d’activité autour des approches dites «de bien-être» en santé publique. Le rapport de 2025 de l’administratrice en chef de la santé publique, *Travailler ensemble pour prospérer: Bien-être et santé publique *(PHAC, 2025), et l’atelier d’été de 2025 et la série de webinaires en cours de l’Association pour la santé publique de la Colombie-Britannique, axés sur les «sociétés du bien-être» (PHABC, s.d.), en sont des exemples récents au Canada.

Le préambule de la Constitution de l’Organisation mondiale de la santé (OMS) de 1946 définit la santé comme étant «un état de complet **bien-être** physique, mental et social [qui] ne consiste pas seulement en une absence de maladie ou d’infirmité» (WHO, s.d., non souligné dans l’original). L’accent sur le bien-être n’a donc rien de nouveau. Comment interpréter ce regain de popularité, et quelles possibilités et limites apporte-t-il à notre vision commune de soutenir la santé publique en cette ère de polycrises?

Il faut situer l’enthousiasme actuel pour le bien-être dans la dynamique internationale croissante autour d’une *économie du bien-être.* Selon l’analyse de la Wellbeing Economy Alliance (créée en 2018), une économie du bien-être – une collaboration de personnes et d’organismes qui travaillent ensemble à transformer le système économique – est:

… une économie au service des gens et de la planète et non l’inverse. Au lieu de traiter la croissance économique comme une fin en soi et de la poursuivre à tout prix, une «économie du bien-être» accorde la primauté aux besoins de l’humanité et de la planète, en s’assurant qu’ils sont pareillement comblés, par défaut. (WEAll, s.d. [traduction libre])

L’élément «économie» est important pour au moins trois raisons. Premièrement, il attire l’attention sur le paradigme du capitalisme néolibéral dominant à l’échelle mondiale, qui alimente les inégalités sociales et les catastrophes économiques (les déterminants sociaux et écologiques de la santé). L’économie politique néolibérale est ancrée dans des modèles économiques qui partent du principe d’une croissance infinie (Ghosh, 2021). Sur une planète aux ressources limitées, ces modèles sont clairement non viables. Pourtant, leur hégémonie entraîne la poursuite continue et de plus en plus agressive d’une vision étroite de la croissance économique par le biais de politiques de déréglementation, d’austérité, de privatisation, de libéralisation des échanges, etc. aux conséquences sociales et écologiques profondément dommageables et inéquitables. Reposant fondamentalement sur la compétition et l’exploitation des humains et de la Terre, ce paradigme s’entrecroise avec le racisme, le sexisme, le capacitisme et d’autres formes de discrimination systémique pour causer des dommages pour la santé très inégaux, et évitables, à toutes les étapes de la vie (McGibbon, 2025). 

Deuxièmement, l’économie du bien-être est importante parce qu’elle nous invite à beaucoup d’égards à nous réapproprier le mot *économie*. Le capitalisme néolibéral est normalisé, ce qui obscurcit les torts immenses qu’il cause. L’affirmation qu’il n’existe aucune autre voie que les budgets d’austérité et les baisses d’impôts est le slogan qui a donné au néolibéralisme sa large légitimité. Dans le contexte canadien, il y a eu des efforts délibérés pour réduire les attentes du public envers le gouvernement et accroître l’apathie politique (Himelfarb, 2024). Dans le discours dominant, «l’économie» passe aussi pour être fixe, extérieure et réservée aux experts (Stanford, 2015). Le cadre et la mouvance de l’économie du bien-être nous invitent plutôt à considérer que «l’économie» est tout bonnement *notre mode de production et de soutien mutuel *ou, encore plus simplement, *la façon dont nous nous occupons les uns des autres* (WEAll, s.d.). L’économie, c’est nous; et le fait de le reconnaître peut renforcer notre pouvoir d’agir.

Troisièmement, et notablement, le cadre de l’économie du bien-être crée l’espace nécessaire pour envisager des options de rechange centrées sur l’équité. Les contours de ces options commencent à se dessiner quand nous nous rendons compte qu’elles sont *aux antipodes* de celles du capitalisme néolibéral. Par exemple, alors que le paradigme économico-politique actuel repose sur la compétition, la rareté et l’exploitation pour organiser la société, les prémisses de rechange, qui viennent de la sagesse et du leadership autochtones, pourraient (ou devraient) inclure l’équité, la collectivité, la réciprocité et la responsabilité les uns envers les autres et envers tout ce qui vit (PHABC, 2026).

Si la santé publique (et l’équité en santé) nous tiennent à cœur, nous devons nous attaquer à ce paradigme économico-politique profondément dommageable, y compris à ceux qui profitent de tels arrangements (McLaren & McGibbon, 2025). Qu’est-ce que les «approches de bien-être» en santé publique ont accompli à cet égard? 

Jusqu’ici, admettons-le, pas grand-chose ou rien du tout. La Charte de Genève pour le bien-être (WHO, 2021), qui aspire aux «sociétés du bien-être», en est une illustration. Faisant fond sur la Charte d’Ottawa pour la promotion de la santé de 1986 et sur les conférences mondiales qui l’ont suivie, la Charte de Genève décrit les «sociétés du bien-être» comme étant celles «qui s’engagent à garantir l’équité en santé aujourd’hui et pour les générations futures sans dépasser les limites écologiques». Il est difficile de contester une telle vision. La Charte est toutefois silencieuse face au paradigme de la croissance économique, bien que ce paradigme soit incompatible avec sa propre vision de l’équité dans les limites écologiques. Remplacer le mot «économie» par la notion plus nébuleuse de «société» risque de plus de diluer ou de détourner notre attention de la portée tentaculaire et indélogeable du capitalisme et de son influence sur tous les aspects de nos vies. 

Une deuxième illustration est le récent guide d’introduction de la Région européenne de l’OMS intitulé *Unlocking Funding for Well-Being, Equity and Healthy Societies *(WHO, 2026), qui non seulement ne remet pas en question le paradigme de la croissance économique, mais l’appuie activement. Il le fait, par exemple, en aspirant à une «croissance économique inclusive» et en cherchant à «équilibrer» divers «capitaux du bien-être», dont le capital humain, social, économique et environnemental. Il est vrai que les gouvernements peuvent faire mieux ou pire pour redistribuer la croissance économique (Osberg, 2021), mais il est parfaitement clair que «la croissance économique à tout prix nous trahit tous et toutes» (De Schutter, 2026). Des expressions comme la «croissance économique inclusive» et la «croissance durable» sont donc contradictoires et doivent être dénoncées. De plus, l’emploi du mot «capital», qui selon la plupart des définitions désigne la richesse monétaire privée, pour décrire les humains et la Terre devrait inciter à une sérieuse réflexion. 

Comment pourrions-nous épouser l’intention radicale du cadre de *l’économie du bien-être*, ou au moins l’envisager sérieusement? Il faut commencer par aborder *d’un œil critique* le capitalisme et le paradigme de la croissance économique, y compris en résistant au cliché qu’il puisse y avoir des arrangements «gagnants-gagnants», bénéfiques à la fois à la santé et à l’économie capitaliste (McLaren & Mykhalovskiy, 2025). Une telle approche est difficile, car elle exige l’humilité de reconnaître que nous sommes nombreux-nombreuses, en santé publique, à profiter de ces structures dommageables à titre de professionnels bien rémunérés et/ou parce que notre domaine de travail – la médecine clinique ou l’épidémiologie, par exemple – est épistémiquement conforme au libéralisme économique, lequel est la source de notre pouvoir et de notre statut. 

Une deuxième façon de nous engager en connaissance de cause est de nous ancrer dans «l’économie», entendue comme étant *notre mode de production et de soutien mutuel, *et de travailler ensemble à définir les contours d’une option de rechange au paradigme actuel, centrée sur l’équité cette fois. La Wellbeing Economy Alliance propose quatre principes pour nous guider (WEAll, s.d.):

***Prédistribution:*** Au lieu de laisser les gens se débrouiller seuls ou dépendre de mécanismes de redistribution limités, nous trouvons des façons de *distribuer au préalable* le pouvoir, la richesse, le temps et les revenus pour que l’économie elle-même fasse le gros du travail.

- Cela nous invite à apprécier les mécanismes autrefois robustes de redistribution par la fiscalité et les transferts du Canada, mis au point sous le régime de l’État providence (et érodés dans une large mesure sous le néolibéralisme), tout en reconnaissant le besoin d’adopter d’autres – et de meilleures – façons de partager le pouvoir en mettant les humains et la planète au premier plan. Les coopératives et les organisations et entreprises détenues par leurs travailleurs, les initiatives de création de richesse communautaire, la normalisation du salaire viable, et l’édification et le renforcement d’économies biorégionales qui incarnent la réciprocité et l’affinité avec la Terre en sont des exemples.

***Raison d’être:*** La raison d’être de l’économie devient exclusivement d’assurer le bien-être humain et écologique.

- Cela comprend d’importantes initiatives en cours pour «mesurer ce qui compte», comme le Cadre de qualité de vie du gouvernement fédéral (Government of Canada, 2025), mais cela va aussi bien plus loin en habilitant le changement de discours essentiel pour que «ce qui compte» *remplace* le PIB et oriente utilement les politiques publiques à différents niveaux.

***Prévention:*** Au lieu de réparer les torts que nous causons à la nature et à l’humanité, nous adoptons des mesures de prévention qui empêchent ces torts de se produire.

- Par exemple, nous continuons de réclamer des démarches réglementaires rigoureuses pour réduire les effets dommageables pour la santé des secteurs d’activité nocifs, comme les combustibles fossiles, l’alimentation et les finances, tout en reconnaissant qu’il est toujours difficile de limiter le pouvoir des entreprises dans une économie qui repose sur la croissance (Puerto, 2026). Nous insistons aussi pour que les services et les mesures de soutien essentiels soient dans le secteur *public* et non dans le privé, pour servir l’intérêt public et non les intérêts d’une minorité privilégiée.

***Action citoyenne:*** Les décisions économiques sont alimentées par les gens, qui participent directement à la prise de décisions et à l’établissement des programmes.

- Cela implique la sensibilisation du public, ainsi que des forums et des espaces pour aider les gens à remplacer l’hégémonie toxique de «l’économie» (fixe, extérieure, exclusiviste) par la prise de conscience que *l’économie, c’est nous*, et que nous pouvons donc la structurer pour qu’elle soit au service de l’humanité et de la planète et non l’inverse. Les assemblées citoyennes et la budgétisation participative (p. ex. CCPA, s.d.) en sont des exemples.

Pour remplacer les principes organisateurs du capitalisme – la compétition, la rareté, la souffrance – par des options de rechange – la collectivité, la réciprocité, la responsabilité – nous pouvons nous inspirer de l’idée de Thurston (brown & Thurston, 2024) de faire du mot «citoyen» un verbe pour favoriser le pouvoir collectif. «Agir en citoyen», selon Thurston, c’est:

- Se manifester, et prendre pour acquis que l’on a un rôle à jouer
- Comprendre ce qu’est le pouvoir et savoir l’exercer
- S’engager en faveur de la collectivité, pas simplement de l’individu
- Investir dans sa relation avec soi-même, les autres et la planète.

Le cadre et la mouvance de *l’économie du bien-être* font donc progresser les approches de «bien-être» à beaucoup d’égards et représentent une magnifique occasion pour les acteurs de la santé publique, quels que soient leurs rôles ou leurs milieux, d’adopter ensemble notre vision commune: soutenir la santé du public. Nous vous invitons cordialement à engager un dialogue permanent et respectueux dans cet espace, y compris (ici je vous tends une perche) en répondant à l’appel d’articles permanent de la Revue sur *Les dimensions écologiques de la santé publique et la société du bien-être *(CJPH, s.d.). 
